# Bacterial-Derived Signals Selectively Remodel Glycosaminoglycan Biosynthetic Pathways in Reconstructed Human Corneal Epithelium

**DOI:** 10.3390/ijms27136046

**Published:** 2026-07-06

**Authors:** Noelia Blanco-Agudín, Natalia Vázquez, Suhui Ye, Cristina Sánchez-Fernández, Iván Fernández-Vega, Álvaro Meana, Jesús Merayo-Lloves, Luis M. Quirós

**Affiliations:** 1 Instituto Universitario Fernández-Vega (IUFV), Fundación de Investigación Oftalmológica, 33006 Oviedo, Spain; blancoanoelia@uniovi.es (N.B.-A.); natalia.vazquez@fio.as (N.V.); yesuhui@uniovi.es (S.Y.); cristina.sanchez@fio.as (C.S.-F.); fernandezvivan@uniovi.es (I.F.-V.); meana@fio.as (Á.M.); 2 Departamento de Biología Funcional, University of Oviedo, 33006 Oviedo, Spain; 3 Instituto de Investigación Sanitaria del Principado de Asturias (ISPA), 33011 Oviedo, Spain; 4 Department of Pathology, Hospital Universitario Central de Asturias (HUCA), 33011 Oviedo, Spain; 5 Biobank of Principality of Asturias, 33011 Oviedo, Spain; 6 Centro Comunitario de Sangre y Tejidos de Asturias, Centro de Investigación Biomédica en Red de Enfermedades Raras (CIBERER U714), 33006 Oviedo, Spain

**Keywords:** corneal epithelium, glycosaminoglycans, bacterial extracellular vesicles, microbial-associated molecular patterns, heparan sulfate, host–microbe interactions

## Abstract

Proteoglycans (PGs) and their glycosaminoglycan (GAG) chains play key roles in corneal epithelial physiology and host–microbe interactions. Although bacterial exposure has been shown to alter PG and GAG biosynthesis, the contribution of specific bacterial-derived signals remains unclear. In this study, reconstructed human corneal epithelia (QobuR) were exposed to bacterial extracellular vesicles (BEVs) from *Pseudomonas aeruginosa* and *Staphylococcus epidermidis*, as well as to lipopolysaccharide, peptidoglycan, and lipoteichoic acid. The expression of 72 genes involved in PG and GAG biosynthesis and remodeling was analyzed by quantitative real-time PCR. Only 22 genes showed significant transcriptional alterations, indicating a highly selective response. Most changes affected enzymes involved in the generation of heparan sulfate (HS) and chondroitin sulfate (CS) fine structure, particularly sulfotransferases. Notably, *HS3ST4* and *HS3ST5* were consistently upregulated under all experimental conditions, suggesting that modulation of HS 3-O-sulfation represents a conserved corneal epithelial response to bacterial-derived stimuli. Whereas microbial-associated molecular patterns induced broader transcriptional responses, BEVs elicited more restricted and species-dependent effects. Overall, these findings demonstrate that bacterial-derived signals selectively remodel GAG biosynthetic pathways and provide new insights into the molecular mechanisms underlying host–microbe interactions at the ocular surface.

## 1. Introduction

The corneal epithelium (CE) constitutes the outermost layer of the cornea and is composed of 5–7 cell layers, including superficial squamous cells, wing cells, and basal cells [1]. Besides contributing to essential ocular functions such as transparency, hydration, and nutrient exchange, CE cells establish tight junctions that form a critical barrier preventing the penetration of pathogens and harmful agents into deeper corneal layers [2,3].

The CE is continuously renewed by limbal epithelial stem cells. Primary epithelial cells isolated from donor tissue retain key in vivo characteristics and can generate stratified epithelial structures with appropriate barrier function under suitable culture conditions [4]. Consequently, several three-dimensional in vitro models have been developed to reproduce the architecture and physiology of native corneal epithelium, providing valuable platforms for studies of corneal biology, toxicity testing, and ophthalmic drug development [5].

Three-dimensional epithelial models reproduce key aspects of the native tissue microenvironment, including cell–cell and cell–matrix interactions that strongly influence cellular behavior and tissue organization [6]. Nevertheless, these systems are generally developed under axenic conditions, whereas the ocular surface is continuously exposed to microorganisms and microbial-derived products originating from the surrounding environment. Although the existence, composition, and functional relevance of a stable corneal microbiota remain subjects of active investigation, increasing evidence indicates that microbial exposure can influence epithelial physiology and immune responses at the ocular surface [7,8]. Proteoglycans (PGs) and their glycosaminoglycan (GAG) chains participate in these host–microbe interactions by acting as receptors involved in microbial adhesion to corneal epithelial cells [9].

PGs are macromolecules composed of a core protein covalently linked to one or more GAG chains. GAGs are linear polysaccharides formed by repeating disaccharide units whose composition varies according to the specific GAG family. These chains undergo extensive post-synthetic modifications, particularly sulfation, generating highly diverse structures responsible for their biological activities [10,11]. Through the selective binding of numerous ligands, including cytokines, chemokines, growth factors, enzymes, enzyme inhibitors, and extracellular matrix proteins [12], PGs regulate essential cellular functions such as cell adhesion and migration, extracellular matrix organization, proliferation, differentiation, morphogenesis, tissue repair, and inflammatory responses [11,13,14]. The structure and biological functions of GAG chains are determined by the coordinated activity of multiple biosynthetic enzymes, whose expression regulates chain composition, sulfation patterns, and ligand-binding properties under physiological and pathological conditions [15].

Recently, a reconstructed human CE model, QobuR, has been developed from primary limbal epithelial cells cultured at the air–liquid interface. This model generates a stratified squamous epithelium that resembles native human CE in its morphological microstructure, barrier properties, and expression of characteristic biomarkers and has been proposed as a platform for applications including preclinical drug screening and ocular hazard assessment [5]. Using this model, our group recently demonstrated that exposure to different bacterial species induces distinct alterations in the expression of genes involved in PG and GAG biosynthesis. Furthermore, controlled exposure to a mixed bacterial consortium representative of microorganisms frequently detected on the ocular surface produced a broader transcriptional response that shifted the expression profile toward that observed in donor-derived corneal epithelia [16].

Our previous studies have also demonstrated that PGs and GAGs act as receptors mediating bacterial adhesion to corneal epithelial cells, following patterns that differ between Gram-positive and Gram-negative microorganisms [9]. However, whether the transcriptional alterations observed following bacterial exposure result from direct bacterial contact or from specific bacterial-derived signals remains unknown. Beyond the role of bacterial adhesins capable of interacting with cellular receptors and GAG chains [17], conserved structural components of bacterial envelopes may contribute to the modulation of epithelial physiology. Among these components, peptidoglycan (PGN), lipopolysaccharide (LPS), and lipoteichoic acid (LTA) are recognized by host pattern-recognition receptors and activate signaling pathways involved in innate immunity, inflammation, and tissue homeostasis [18,19].

In addition, both Gram-positive and Gram-negative bacteria release bacterial extracellular vesicles (BEVs), nanosized structures involved in host–microbe communication. BEVs transport a complex cargo of proteins, lipids, nucleic acids, and other bioactive molecules whose composition depends on the bacterial species and environmental conditions and are capable of modulating host-cell signaling pathways and gene expression [20,21,22,23].

While the role of PGs and GAGs in bacterial adhesion to corneal epithelial cells, as well as the modulation of their biosynthetic pathways following bacterial exposure, has been previously established, little is known about how bacterial-derived signals influence the biosynthetic pathways responsible for their synthesis and structural diversification. Given the central role of these molecules in epithelial physiology and host–microbe interactions, understanding how bacterial structural components and BEVs modulate their expression may provide new insights into the mechanisms governing corneal epithelial homeostasis. Therefore, in the present study, we analyzed the transcriptional profiles of genes involved in PG and GAG biosynthesis in the QobuR model following exposure to BEVs produced by *Pseudomonas aeruginosa* and *Staphylococcus epidermidis*, as well as to microbial-associated molecular patterns (MAMPs), including LPS, PGN, and LTA. Our results demonstrate that both BEVs and bacterial structural components induce distinct yet partially overlapping transcriptional responses, predominantly targeting enzymes responsible for the generation of the fine structure of heparan sulfate (HS) and chondroitin sulfate (CS) chains.

## 2. Results

### 2.1. Bacterial Extracellular Vesicles and MAMPs Induce Distinct Transcriptional Signatures in Genes Involved in GAG Biosynthesis

Transcripts corresponding to most of the 72 genes analyzed were readily detected in reconstructed corneal epithelia (Supplementary Figure S1). Exposure of the CE model to bacterial-derived stimuli, including BEVs and MAMPs, induced significant alterations in the expression of specific genes involved in PG and GAG biosynthesis. The transcriptional responses observed were highly dependent on the nature of the bacterial stimulus.

**Figure 1:**
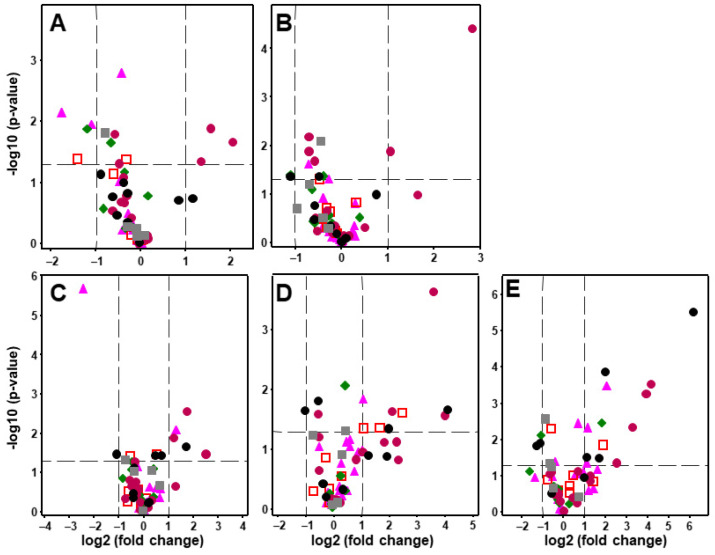
Volcano plots representing differential transcriptional analysis of PG- and GAG-coding genes induced by exposure to bacterial-derived stimuli. The threshold in the volcano plot was *p*-value < 0.05 and |log_2_ fold change| > 1. (**A**) extracellular vesicles from *S. epidermidis*; (**B**) extracellular vesicles from *P. aeruginosa*; (**C**) peptidoglycan; (**D**) lipopolysaccharide; (**E**) lipoteichoic acid. Different gene groups are identified by distinct symbols: 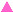, PGs; 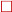, HS/CS tetrasaccharide linker; 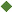, GAG glycosyltransferases; 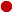, fine structure synthesis of HS; 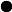, fine structure synthesis of CS; 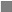, hydrolases.

Exposure to BEVs derived from *S. epidermidis* and *P. aeruginosa* resulted in a limited number of significant transcriptional changes (Figure 1A,B; Supplementary Figure S1). Both types of BEVs induced downregulation of *CHSY3*, encoding a glycosyltransferase involved in CS biosynthesis, together with increased expression of *HS3ST4* and *HS3ST5*, two enzymes responsible for the 3-O-sulfation of glucosamine residues within HS chains. In addition, *P. aeruginosa* BEVs specifically reduced *CHST15* expression, whereas *S. epidermidis* BEVs increased *HS6ST3* expression and decreased transcript levels of the PGs *SDC2* and *SRGN* (Figure 1A,B).

In contrast, exposure to MAMPs elicited broader transcriptional responses, predominantly characterized by gene upregulation and largely dependent on the molecule tested (Figure 1C–E; Supplementary Figure S1). PGN mainly affected enzymes involved in HS modification, increasing the expression of *HS3ST3A1*, *HS3ST4*, and *HS3ST5*. In addition, PGN upregulated *CHST13* and the PG *GPC2*, while reducing the expression of the CS-modifying enzyme *CHST15* and the PG *SRGN* (Figure 1C).

LPS exposure induced the upregulation of the PG *COL18A1*, three genes involved in HS/CS linkage tetrasaccharide synthesis (*B3GALT6*, *B3GAT3*, and *PXYLP1*), the HS-modifying enzymes *HS6ST3*, *HS3ST4*, and *HS3ST5*, and the CS-modifying enzymes *CHST12* and *CHST13*. In contrast, expression of the CS-modifying enzyme *UST* was reduced (Figure 1D).

Among all conditions tested, LTA induced the greatest number of transcriptional alterations, most of which corresponded to gene upregulation. Significant increases were observed in transcripts encoding the PGs *SDC3*, *GPC2*, and *COL18A1*; the glycosyltransferases *B3GALT6* and *CHSY3*; four *HS3ST* isoforms (*HS3ST2*, *HS3ST3A1*, *HS3ST4*, and *HS3ST5*); and four enzymes involved in CS sulfation (*CHST12*, *CHST13*, *CHST14*, and *CHST3*). Conversely, the expression of *EXT2*, *DSE*, and *UST* was significantly reduced (Figure 1E).

Notably, *HS3ST4* and *HS3ST5* were consistently upregulated under all experimental conditions, identifying HS 3-O-sulfation as the GAG modification most consistently affected by bacterial-derived stimuli.

### 2.2. Transcriptional Alterations Affect a Limited Subset of Genes and Preferentially Target Specific Biosynthetic Steps

**Figure 2:**
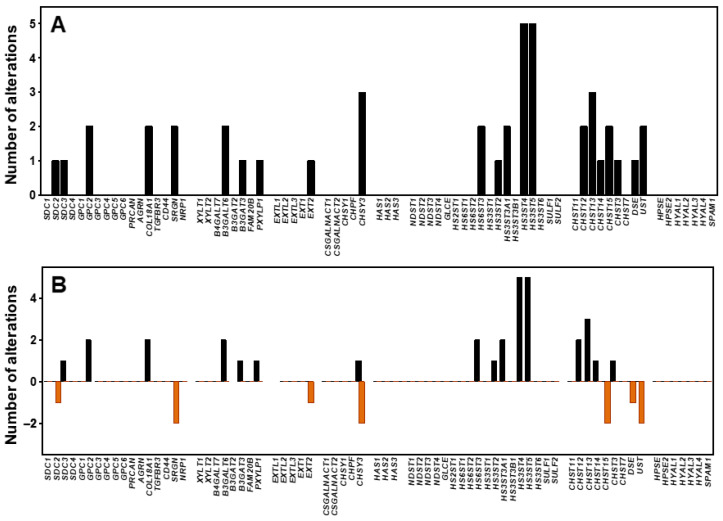
Number of expression changes detected for each analyzed gene. (**A**) Total changes; histogram bars indicate the number of experimental conditions, among all those tested, in which each gene showed altered expression. (**B**) Over- and underexpression; positive bars indicate the number of conditions in which each gene was overexpressed, whereas negative bars indicate the number of conditions in which it was underexpressed.

Only 22 of the 72 genes analyzed exhibited significant transcriptional alterations under at least one of the experimental conditions tested, representing 31% of the total gene set examined (Figure 2A). Most of these changes corresponded to gene upregulation (65%), whereas downregulation accounted for approximately 35% of the detected alterations (Figure 2B).

Interestingly, the direction of regulation remained remarkably consistent across stimuli. Genes that were upregulated under one condition were never found to be downregulated under another, and vice versa. The only exception was *CHSY3*, whose transcript levels decreased following exposure to BEVs but increased after treatment with LTA (Figure 2B).

Most genes were altered under only one or two experimental conditions, accounting for 9 of the 22 altered genes in each case. In contrast, *CHSY3* and *CHST13* were modified under three different conditions, whereas *HS3ST4* and *HS3ST5* were altered in all five experimental situations analyzed (Figure 2A).

Importantly, the observed changes were not randomly distributed throughout the biosynthetic pathways. More than 70% of the altered genes were associated with the synthesis and structural modification of HS and CS chains. Moreover, the majority of alterations preferentially affected genes encoding sulfotransferases involved in the generation of the fine structure of both GAGs, accounting for more than 60% of all changes detected (34% and 27% corresponding to HS- and CS-related sulfotransferases, respectively).

**Figure 3:**
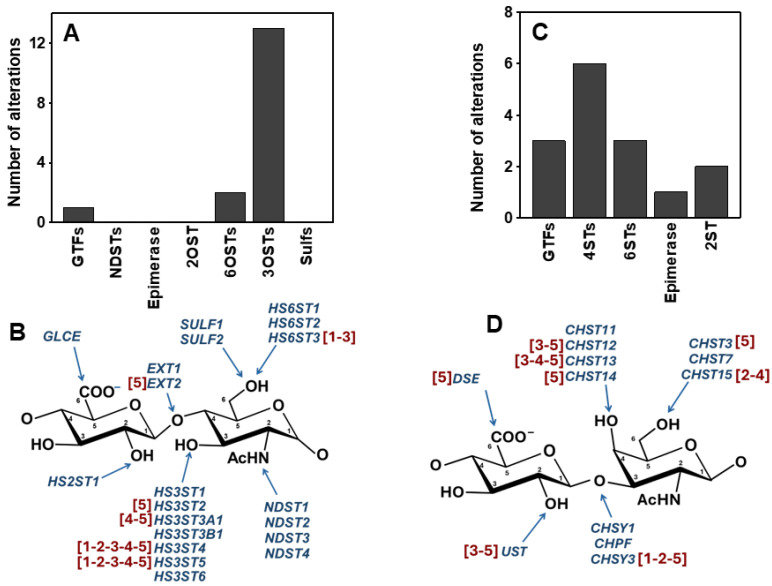
Transcriptional alterations observed in genes involved in HS and CS chain biosynthesis. (**A**) Total transcriptional changes in genes involved in each stage of HS chain biosynthesis. (**B**) HS disaccharide unit indicating the genes involved at each biosynthetic step, with treatment conditions showing significant alterations highlighted in red. (**C**) Total transcriptional changes in genes involved in each stage of CS chain biosynthesis. (**D**) CS disaccharide unit indicating the genes involved at each biosynthetic step, with treatment conditions showing significant alterations highlighted in red. Numbers represent: 1, extracellular vesicles from *S. epidermidis*; 2, extracellular vesicles from *P. aeruginosa*; 3, lipopolysaccharide; 4, peptidoglycan; 5, lipoteichoic acid.

This pattern was particularly evident in the HS pathway, where transcriptional alterations were concentrated in genes encoding enzymes involved in the terminal sulfation of glucosamine residues, especially members of the *HS3ST* family (Figure 3A,B). Among them, *HS3ST4* and *HS3ST5* emerged as the most consistently affected genes, displaying increased expression under all experimental conditions tested.

Alterations affecting CS biosynthesis were more broadly distributed across the pathway and involved enzymes participating in multiple biosynthetic reactions. Nevertheless, genes encoding sulfotransferases responsible for C4 sulfation of galactosamine residues represented the largest fraction of CS-related changes (Figure 3C,D).

In contrast, genes involved in core protein synthesis and chain initiation or elongation were only rarely affected. Notably, no transcriptional alterations were detected in genes encoding enzymes responsible for hyaluronic acid biosynthesis. Likewise, genes involved in GAG degradation, including heparanases and hyaluronidases, remained completely unaltered under all experimental conditions analyzed.

Taken together, these findings indicate that bacterial-derived stimuli do not induce a global remodeling of PG/GAG biosynthetic or degradation pathways. Instead, they preferentially target the terminal enzymatic steps responsible for the fine structural diversification of HS and CS chains.

### 2.3. Bacterial-Derived Stimuli Induce Distinct but Partially Overlapping Transcriptional Responses

**Figure 4:**
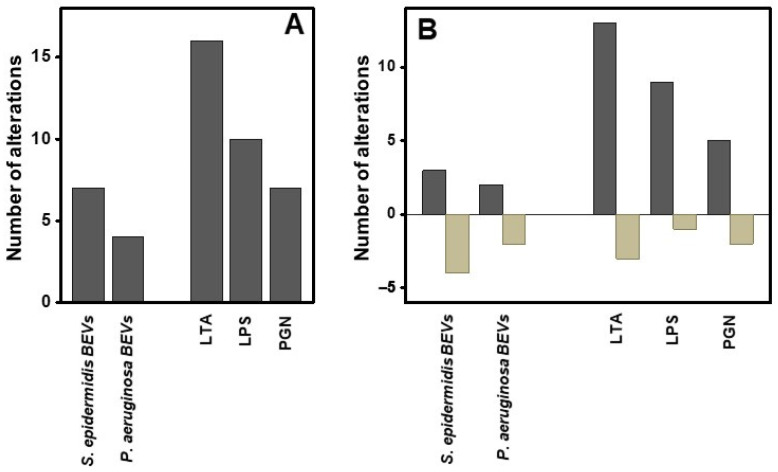
Transcriptional alterations induced by extracellular vesicles and microbial molecules. (**A**) Total number of genes with altered transcription under each experimental condition. (**B**) Up- and downregulated genes; dark bars indicate the number of genes with increased transcription, whereas light bars indicate the number of genes with decreased transcription.

The number of transcriptional alterations varied considerably among experimental conditions. BEVs derived from *S. epidermidis* induced nearly twice as many changes as those produced by *P. aeruginosa* (seven versus four alterations, respectively; Figure 4A). In both cases, gene downregulation represented a substantial proportion of the response, accounting for 50% of the alterations induced by *P. aeruginosa* BEVs and 57% of those induced by *S. epidermidis* BEVs (Figure 4B).

More pronounced effects were observed following exposure to MAMPs. PGN induced seven significant transcriptional alterations, whereas LPS and, particularly, LTA triggered broader responses affecting 10 and 16 genes, respectively (Figure 4A). In all three cases, gene upregulation predominated, accounting for approximately 70–80% of the transcriptional changes detected (Figure 4B).

**Figure 5:**
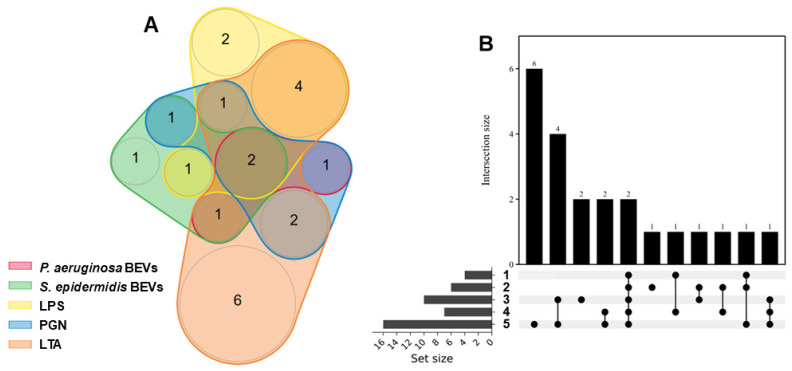
Diagrams showing shared transcriptional alterations among different experimental conditions. (**A**) Venn diagram, where the colors of each region indicate the different experimental conditions and numbers indicate genes included in each individual or shared region. (**B**) UpSet plot displaying intersections of differentially expressed genes across conditions. The *y*-axis represents the number of altered genes in each set or intersection, and the *x*-axis represents the corresponding condition combinations. Numbers indicate the different treatment conditions: 1, *P. aeruginosa* BEVs; 2, *S. epidermidis* BEVs; 3, lipopolysaccharide; 4, peptidoglycan; 5, lipoteichoic acid.

Relationships among experimental conditions were further explored by intersection analyses (Figure 5A,B). Two genes, *HS3ST4* and *HS3ST5*, were consistently altered under all experimental conditions tested. LTA exhibited the highest number of unique transcriptional changes (six genes), followed by LPS with two condition-specific alterations. The strongest overlap in transcriptional responses was observed between LTA and LPS, which shared four altered genes, whereas only two genes were common to PGN and LTA. In all other cases, overlap was minimal, with only a single gene shared among two or three experimental conditions.

Together, these findings indicate that bacterial-derived stimuli do not induce random remodeling of PG- and GAG-related pathways. Instead, they generate reproducible and partially overlapping transcriptional responses centered on a limited set of genes. In particular, *HS3ST4* and *HS3ST5* emerged as common targets of both BEVs and MAMPs, identifying the 3-O-sulfation of HS chains as the modification most consistently influenced by bacterial-derived stimuli.

### 2.4. Lipopolysaccharide and Lipoteichoic Acid Induce the Greatest Global Transcriptional Divergence, Whereas BEV-Treated Epithelia Remain Closer to the Basal QobuR Profile

**Figure 6:**
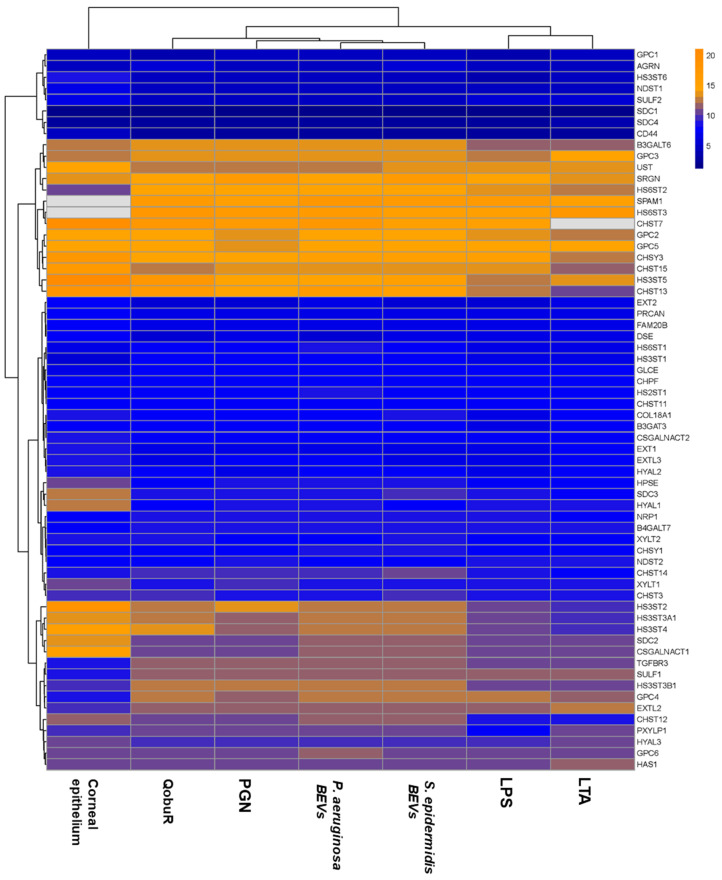
Heatmap and hierarchical clustering of PG- and GAG-related gene transcription across all experimental conditions and donor corneal epithelium. Analyses were performed using normalized qPCR expression values (ΔCt values). Color scale represents normalized ΔCt values.

To determine whether bacterial-derived stimuli altered the overall transcriptional landscape of reconstructed corneal epithelia, hierarchical clustering analysis was performed using normalized ΔCt values obtained for all PG- and GAG-related genes analyzed (Figure 6).

All experimental conditions retained a global expression pattern broadly similar to that observed in untreated QobuR, indicating that bacterial-derived stimuli affected only a limited subset of genes. Nevertheless, clear differences among treatments were evident. The transcriptional profiles induced by *P. aeruginosa* and *S. epidermidis* BEVs clustered closely together and remained highly related to untreated QobuR, suggesting that vesicle-induced changes were relatively restricted at the global level. Similarly, PGN-treated samples grouped within this cluster and showed a high degree of similarity to untreated QobuR (Figure 6).

In contrast, LPS- and LTA-treated epithelia formed a distinct subgroup separated from the remaining experimental conditions, indicating that these molecules induced the greatest remodeling of PG- and GAG-related gene expression. This observation is consistent with the larger number of transcriptional alterations detected for both stimuli, particularly in response to LTA (Figure 4).

Comparison with donor-derived corneal epithelia revealed that native tissue displayed a differentiated transcriptional profile relative to QobuR and all treated conditions. However, the clustering pattern indicated that exposure to bacterial-derived stimuli did not shift the reconstructed epithelium toward a completely distinct transcriptional state. Rather, the observed changes occurred within a conserved PG/GAG expression framework in which only a limited number of genes were selectively modified.

Because the hierarchical clustering analysis was based on normalized ΔCt values rather than fold changes relative to untreated controls, the relationships observed reflect overall transcriptional similarities among experimental groups and donor-derived corneal epithelia, rather than differential expression patterns derived from individual gene fold-change analyses.

## 3. Discussion

Our previous work demonstrated that direct exposure of reconstructed corneal epithelia to different bacterial species selectively alters the expression of genes involved in PG and GAG biosynthesis, particularly those responsible for generating the fine structural features of HS and CS [16]. The present study extends these observations by demonstrating that bacterial-derived signals alone, including BEVs and conserved MAMPs, are sufficient to induce this selective transcriptional remodeling. These findings indicate that direct bacterial contact is not required to trigger the changes observed in PG and GAG biosynthetic pathways and suggest that soluble microbial signals constitute key mediators of corneal epithelial glycocalyx remodeling.

In the present study, we sought to determine whether bacterial-derived signals alone are sufficient to induce transcriptional remodeling of PG and GAG biosynthetic pathways. Our results demonstrate that both BEVs and conserved bacterial structural components trigger significant, although selective, alterations in the expression of genes involved in PG and GAG biosynthesis.

A first notable observation is that only a minority of the genes analyzed were affected. Approximately 70% of the genes examined remained unchanged under all experimental conditions, indicating that bacterial-derived stimuli do not induce a generalized dysregulation of PG and GAG biosynthesis. Instead, the response was highly selective and focused on a limited number of targets. This observation is consistent with current concepts regarding GAG biology, according to which major functional changes can arise from modifications affecting only a small subset of biosynthetic enzymes, particularly those involved in generating the fine structural features responsible for ligand recognition [10,13].

Indeed, the most remarkable finding of this study was the recurrent alteration of genes involved in the final stages of GAG biosynthesis, particularly those involved in generating HS fine structure. This observation is especially relevant considering that HS is the predominant GAG present at the cell surface and within the pericellular region [11], where it serves as a major regulator of interactions with cytokines, chemokines, growth factors, extracellular matrix components, and numerous other signaling molecules [12]. In both HS and CS biosynthetic pathways, most transcriptional changes affected sulfotransferases responsible for terminal sulfation reactions rather than enzymes involved in initial chain assembly. This pattern closely resembles that observed previously following direct bacterial exposure [16], suggesting that bacterial-derived signals preferentially target the generation of structural diversity rather than the synthesis of the polysaccharide backbone itself.

In the case of HS, particularly striking was the consistent overexpression of *HS3ST4* and *HS3ST5*, which occurred under all five experimental conditions examined. These genes encode members of the HS3ST family, enzymes responsible for the 3-O-sulfation of glucosamine residues within HS chains, representing the final sulfation step during HS biosynthesis. Although 3-O-sulfation affects only a minor fraction of HS disaccharides, it generates highly specialized binding domains that mediate selective interactions with a restricted repertoire of ligands [24]. The fact that *HS3ST4* and *HS3ST5* represented the only common transcriptional signature shared by all bacterial-derived stimuli, together with our previous study demonstrating that these same genes were likewise consistently upregulated following direct exposure of reconstructed CE to intact bacteria [16], strongly suggests that modulation of HS 3-O-sulfation may represent a conserved epithelial transcriptional response to microbial challenge. Similar observations have been reported in other host–microorganism systems, where microbial interactions alter the expression of genes involved in HS fine structure, supporting the concept that these modifications contribute to epithelial adaptation to microbial exposure [17].

Despite the remarkable consistency of this response, the functional consequences of increased *HS3ST4* and *HS3ST5* expression remain to be established. Unlike most GAG modifications, 3-O-sulfation generates rare and highly specific structural motifs within HS chains rather than producing global changes in HS abundance. These specialized domains have been implicated in the selective regulation of certain protein–HS interactions, suggesting that even subtle changes in 3-O-sulfation could have important biological consequences. Moreover, because 3-O-sulfated motifs are of very low abundance and reliable experimental tools for their direct detection and functional characterization remain limited, establishing a causal relationship between the transcriptional changes observed here and specific epithelial functions remains technically challenging. Future studies combining glycomic profiling of HS chains, selective detection of 3-O-sulfated domains, and targeted perturbation of *HS3ST4* and *HS3ST5* expression will therefore be required to determine how microbial modulation of HS 3-O-sulfation influences corneal epithelial biology. Such approaches were beyond the scope of the present study but represent an important direction for future research aimed at understanding how microbial signals remodel the corneal epithelial glycocalyx.

Alterations affecting CS biosynthesis displayed a distinct but conceptually similar pattern. As observed for HS, most transcriptional changes involved enzymes responsible for generating chain fine structure rather than those involved in core polymer assembly. Notably, these alterations were predominantly induced by MAMPs and were largely absent following BEV exposure. More than half of the altered CS-related genes encoded sulfotransferases involved in the 4-O-sulfation of N-acetylgalactosamine residues, the modification responsible for generating CS-A domains, one of the most abundant sulfation motifs in mammalian tissues [25].

Although CS chains are less abundant than HS within the epithelial glycocalyx and pericellular matrix and interact with a comparatively narrower range of ligands, they nevertheless play important roles in the regulation of multiple cellular processes, including extracellular matrix remodeling, inflammatory responses, cell migration, and tissue homeostasis [11,12]. Therefore, the increase in genes associated with CS 4-sulfation observed here may indicate a tendency toward selective remodeling of CS fine structure that could contribute to the modulation of inflammatory signaling following exposure to bacterial components. Interestingly, this response was primarily associated with purified bacterial molecules, suggesting that activation of pattern-recognition receptors may preferentially influence CS sulfation pathways. The absence of a comparable response in BEV-treated epithelia is consistent with the notion that vesicle-associated signals activate regulatory programs that differ from those triggered by isolated pathogen-associated molecular patterns.

An additional finding of interest concerns the differences observed between BEVs and MAMPs. Whereas MAMPs induced broader and partially overlapping transcriptional responses, BEVs elicited comparatively fewer alterations but generated distinct expression profiles. Hierarchical clustering and intersection analyses further revealed a closer relationship between LPS- and LTA-induced responses than between either of them and BEV-induced responses. These observations are consistent with the activation of conserved innate immune pathways by classical pathogen-associated molecular patterns, which engage pattern-recognition receptors and converge on common downstream signaling cascades, including NF-κB and MAPK signaling [18,19].

In contrast, BEVs elicited comparatively limited transcriptional responses but generated distinct expression profiles that differed from those induced by purified bacterial molecules. Rather than reproducing the effects of individual MAMPs, BEVs deliver complex combinations of proteins, lipids, nucleic acids, polysaccharides, and other bioactive molecules whose composition varies according to bacterial species and environmental conditions [20,21,22,23]. Consequently, despite inducing fewer transcriptional alterations, BEVs may act as selective and species-dependent signaling platforms that modulate specific host pathways not activated by individual bacterial structural components alone. This interpretation is supported by the existence of exclusive alterations observed in BEV-treated samples and by their distinct clustering patterns relative to the responses induced by LPS and LTA. Interestingly, the predominance of alterations affecting sulfotransferases rather than polymerizing enzymes closely mirrors the transcriptional profile previously observed following direct bacterial exposure [16], suggesting that modulation of GAG fine structure represents a common epithelial strategy in response to both intact microorganisms and bacterial-derived signals.

Importantly, although several transcriptional alterations induced by bacterial-derived stimuli overlapped with those previously observed following direct bacterial exposure, none of the individual stimuli reproduced the complete pattern generated by intact microorganisms [16]. This observation suggests that the epithelial response to bacteria results from the integration of multiple simultaneous signals, including microbial adhesion, recognition of conserved microbial molecules, and vesicle-mediated communication. Such a multifactorial response is likely to better reflect the complexity of host–microorganism interactions occurring at exposed epithelial surfaces.

Hierarchical clustering analysis further indicated that all experimental conditions remained globally similar to untreated QobuR, despite the significant alterations observed in specific genes. Thus, bacterial-derived stimuli induced selective remodeling within an otherwise stable transcriptional framework. The most divergent profiles were observed following exposure to LPS and LTA, whereas BEV-treated epithelia remained closer to the basal state. These observations reinforce the notion that the principal effect of bacterial-derived stimuli is the modulation of discrete regulatory nodes within PG and GAG biosynthetic pathways rather than widespread transcriptional reprogramming.

Several limitations should be acknowledged. The present study focused primarily on transcriptional changes and did not directly evaluate the resulting structural modifications of HS or CS chains. Although the relationship between gene expression and the final GAG architecture is complex and not yet fully understood, previous studies have shown that the expression of biosynthetic and modifying enzymes constitutes a major determinant of GAG composition and sulfation patterns. Therefore, the transcriptional alterations reported here may reflect biologically relevant remodeling processes. In addition, because the microbial-derived molecules investigated differ in molecular structure, receptor usage, and biological activity, the concentrations employed should not be interpreted as biologically equivalent doses but rather as experimental conditions selected within previously reported ranges for epithelial models and applied consistently across all experimental groups. Future studies combining transcriptomic analyses with disaccharide composition, sulfation profiling, and functional assays will nevertheless be required to establish the precise structural and functional consequences of these changes.

In addition, the present work evaluated BEVs produced by two clinically relevant ocular microorganisms representing distinct host–microbe interactions: the opportunistic pathogen *P. aeruginosa*, the leading cause of bacterial keratitis, and the commensal species *S. epidermidis*, one of the predominant members of the healthy ocular surface microbiota. Likewise, three MAMPs associated with both Gram-positive and Gram-negative bacteria were examined. Although these models encompass major pathogenic and commensal stimuli at the ocular surface, additional microorganisms and microbial factors may induce distinct GAG responses and should be investigated in future studies.

## 4. Materials and Methods

### 4.1. Microorganisms and Culture Media

The bacterial species used in this study were *Pseudomonas aeruginosa* and *Staphylococcus epidermidis*, both obtained from clinical isolates provided by the Central University Hospital of Asturias (Oviedo, Spain). Bacteria were routinely cultured in Brain Heart Infusion Broth (BHI) (Merck, Sigma-Aldrich, St. Louis, MO, USA) at 37 °C under shaking conditions until reaching the exponential growth phase, after which cultures were processed for BEV isolation.

### 4.2. Development of Reconstructed Corneal Epithelium Models

Human donor corneal tissues were handled in accordance with the Declaration of Helsinki. Three human donor corneas without known ocular surface disease were obtained from the Fernández-Vega Ophthalmological Institute (Oviedo, Asturias, Spain) from tissue remaining after penetrating keratoplasty procedures. All tissues were stored at 4 °C in Eusol-C medium (Alchimia, Ponte San Nicolò, Italy) for no longer than 10 days before use.

Reconstructed CE (QobuR) models were generated from limbal epithelial cells according to a previously described protocol [5].

### 4.3. Isolation of Corneal Epithelia from Donor Corneas

Human donor corneal tissues were handled in accordance with the principles of the Declaration of Helsinki. Three human donor corneas without known ocular surface disease, discarded for transplantation exclusively because of low endothelial cell density, were obtained from the Community Center for Blood and Tissue (Oviedo, Asturias, Spain). Briefly, the corneal surface was gently scraped from the center to the periphery using a fine needle, and the epithelium was collected and transferred into tubes containing sterile phosphate-buffered saline (PBS). Samples were immediately processed for RNA isolation or stored at −80 °C until use.

### 4.4. Isolation and Quantification of Bacterial Extracellular Vesicles

BEVs produced by *P. aeruginosa* and *S. epidermidis* were isolated following protocols previously described with minor modifications [26,27]. Briefly, bacterial cultures grown to an optical density at 600 nm (OD600) of 0.5 were diluted 1:100 in fresh BHI medium and incubated for 24 h at 37 °C under shaking conditions.

Cultures were centrifuged at 6000 rpm for 20 min at 4 °C, followed by a second centrifugation at 10,000 rpm under the same conditions. Supernatants were subsequently filtered through 0.22 μm pore-size filters (Sigma-Aldrich, St. Louis, MO, USA) to ensure complete bacterial removal. Sterility controls were performed by plating 100 μL of each filtrate onto BHI agar plates. Filtered supernatants were concentrated by ultrafiltration at 4700× *g* for 15 min using 100 kDa molecular weight cut-off PES protein concentrators (Thermo Scientific Pierce, Waltham, MA, USA). BEVs were then pelleted by ultracentrifugation at 100,000× *g* for 2.5 h using a Beckman SW 60 Ti rotor (Beckman Coulter, Brea, CA, USA). The resulting pellets were resuspended in phosphate-buffered saline (PBS), washed by a second ultracentrifugation step under identical conditions, and finally resuspended in PBS. Purified BEVs were stored at −20 °C until use.

BEV concentration and size distribution were determined by nanoparticle tracking analysis (NTA) using a NanoSight LM10 instrument (Malvern Panalytical, Malvern, UK), as previously described [28]. Representative transmission electron microscopy (TEM) images illustrating the characteristic morphology and nanoscale size of the isolated BEVs were acquired following the protocol described in [28] and are provided in Supplementary Figure S2.

### 4.5. Exposure of Reconstructed Corneal Epithelia to Bacterial-Derived Stimuli

Reconstructed corneal epithelia were generated in 1.12 cm^2^ Transwell^®^ inserts and exposed to bacterial-derived stimuli as described below. Depending on the experiment, epithelia were exposed either to BEVs or to purified bacterial molecules. In all cases, treatments were carried out for 15 h at 37 °C in a humidified atmosphere containing 5% CO_2_.

For BEV exposure experiments, BEVs were added at a ratio of 1000 vesicles per epithelial cell in a final volume of 500 μL of phosphate-buffered saline (PBS).

For experiments involving bacterial structural components, LPS from *Escherichia coli* O111 (Merck, Sigma-Aldrich, St. Louis, MO, USA), PGN from *Staphylococcus aureus* (Merck, Sigma-Aldrich, St. Louis, MO, USA), and LTA from *Staphylococcus aureus* (Merck, Sigma-Aldrich, St. Louis, MO, USA) were added at final concentrations of 1 ng/mL, 5 μg/mL, and 50 μg/mL, respectively. The concentrations of the different microbial-associated molecular patterns (LPS, PGN and LTA) were selected based on concentrations previously reported to induce reproducible epithelial responses in ocular and other epithelial cell models while preserving cell viability and were maintained throughout the study to facilitate comparison among the different microbial-derived stimuli [29,30,31].

### 4.6. RNA Isolation, cDNA Synthesis and qRT-PCR Analysis

Total RNA was isolated using the RNeasy Mini Kit (Qiagen, Hilden, Germany) according to the manufacturer’s instructions. Prior to RNA extraction, reconstructed corneal epithelia were washed three times with 500 μL of phosphate-buffered saline (PBS), and cells were lysed directly in the inserts using 200 μL of RLT buffer per well.

For donor-derived corneal epithelia, samples were resuspended in 600 μL of RLT buffer and homogenized using a Polytron PT 2100 homogenizer (Kinematica Inc., Bohemia, NY, USA) for 1 min at 30,000 rpm, followed by centrifugation at 15,000× *g* to remove cellular debris.

To eliminate residual genomic DNA contamination, RNA samples were treated with RNase-Free DNase (Qiagen) during the purification procedure. cDNA synthesis was performed using the High-Capacity cDNA Reverse Transcription Kit (Applied Biosystems, Foster City, CA, USA) according to the manufacturer’s instructions, as previously described [32].

qRT-PCR reactions, amplification conditions and data analyses were performed as previously described [32]. Primer sequences are listed in Supplementary Table S1, together with the corresponding Gene IDs and encoded proteins for all genes analyzed. Glyceraldehyde-3-phosphate dehydrogenase (GAPDH) was used as the reference gene for normalization of gene expression levels.

### 4.7. Statistical Analysis and Graphical Representation

The Mann–Whitney U test was used to compare differences between experimental groups. For qPCR analyses, three CE models derived from independent donors were used, with each reaction performed in at least triplicate for each donor and additional replicates included when required. Cluster analysis was performed using the Statistics for Windows software, Version 12 (StatSoft, Inc., Tulsa, OK, USA). Heatmaps were generated using R Statistical Computing, Version 4.4.1 (R Foundation for Statistical Computing, Vienna, Austria). For hierarchical clustering analyses, normalized ΔCt values rather than fold changes relative to the control condition were used. Consequently, clustering analyses and heatmaps reflect overall expression similarities among experimental conditions and donor-derived corneal epithelia rather than differential expression relative to a specific control group. Venn diagrams were created with the nVenn tool (http://degradome.uniovi.es/cgi-bin/nVenn/nvenn.cgi (accessed on 6 April 2026)) [33], and UpSet plots were generated using the software available at https://www.chiplot.online/upset_plot.html (accessed on 6 April 2026).

## 5. Conclusions

Our results demonstrate that BEVs and MAMPs are sufficient to induce selective remodeling of genes involved in PG and GAG biosynthesis in reconstructed corneal epithelia. These responses preferentially affect enzymes responsible for generating the fine structural features of HS and CS chains, particularly those catalyzing terminal sulfation reactions. MAMPs induced partially overlapping transcriptional programs, whereas BEVs elicited more species-dependent responses. Among all genes analyzed, HS3ST4 and HS3ST5 emerged as common targets of all bacterial-derived stimuli, suggesting that modulation of HS 3-O-sulfation may represent a conserved epithelial response to microbial exposure. Overall, these findings highlight the importance of bacterial-derived signals in shaping corneal epithelial glycocalyx composition and provide new insights into the molecular mechanisms underlying host–microbe interactions at the ocular surface.

## Data Availability

The original contributions presented in the study are included in the article/Supplementary Material. Further inquiries can be directed to the corresponding authors.

## Supplementary Materials

The following supporting information can be downloaded at: https://www.mdpi.com/article/10.3390/ijms27136046/s1.

## Abbreviations

The following abbreviations are used in this manuscript:

BEV	Bacterial extracellular vesicle
CE	Corneal epithelium
CS	Chondroitin sulfate
GAG	Glycosaminoglycan
HS	Heparan sulfate
LPS	Lipopolysaccharide
LTA	Lipoteichoic acid
MAMPs	Microbial-associated molecular patterns
PG	Proteoglycan
PGN	Peptidoglycan
