# Supplementary material for: Bacterial-Derived Signals Selectively Remodel Glycosaminoglycan Biosynthetic Pathways in Reconstructed Human Corneal Epithelium
Source: Int J Mol Sci. 2026 Jul 6;27(13):6046. doi: 10.3390/ijms27136046 (PMC13361807; doi:10.3390/ijms27136046)
Supplement: Supplementary file 1 [file ijms-27-06046-s001.zip › ijms-4413652-supplementary.pdf]

Supplementary Figure S1

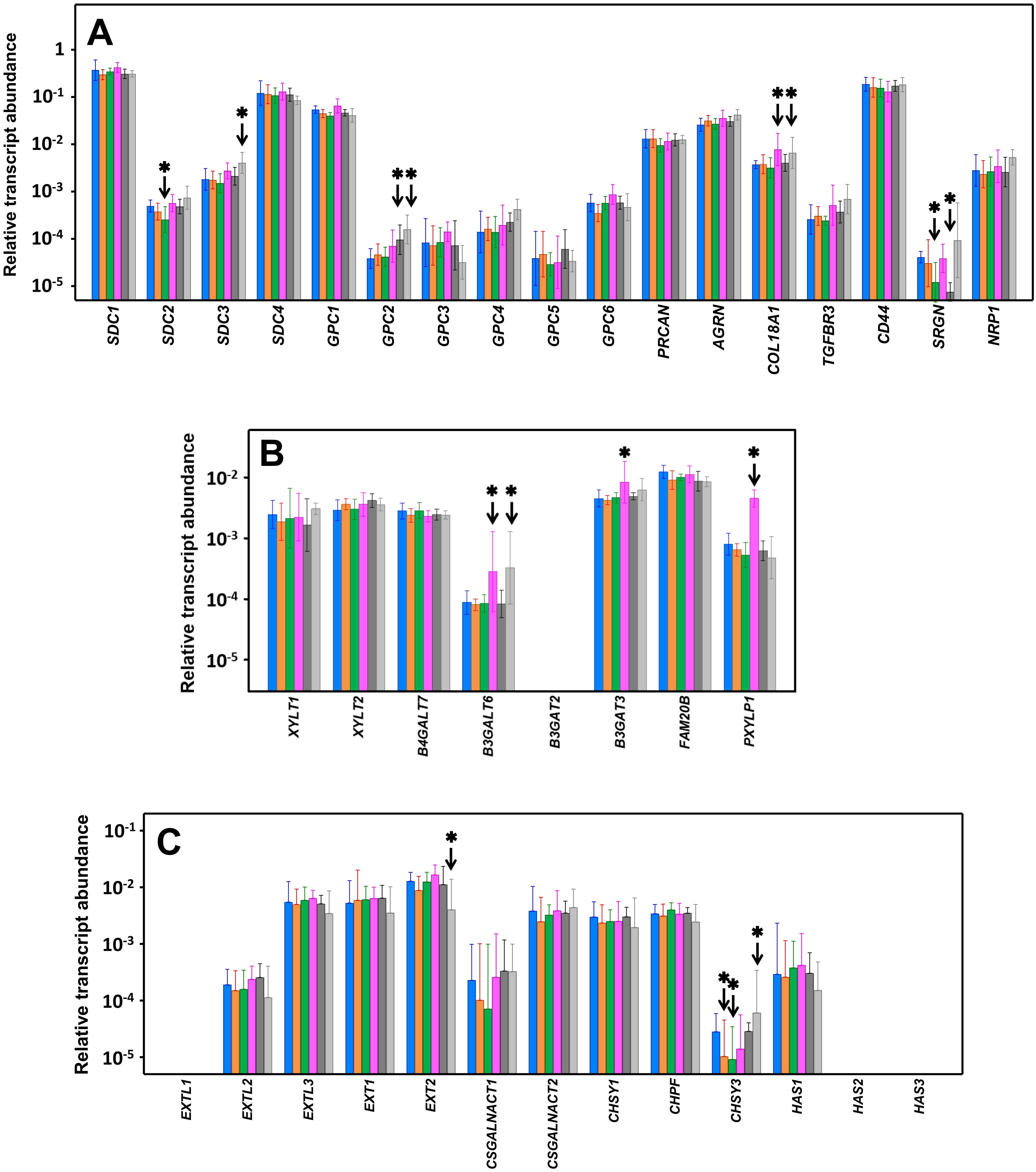

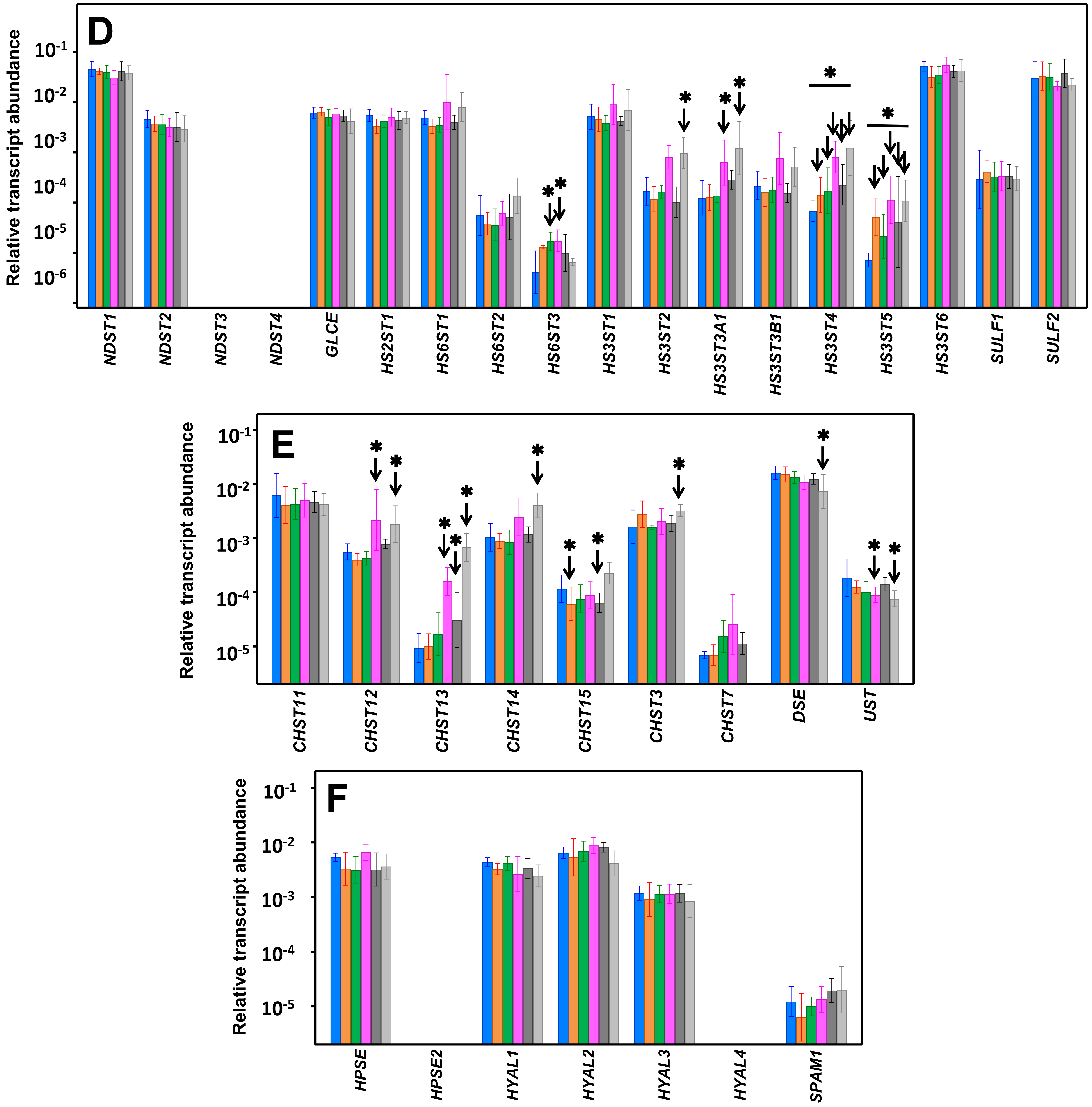

Relative transcript abundance of genes encoding PGs and GAGs in reconstructed corneal epithelium.
Relative transcript abundance of mRNAs is shown for:
A, proteoglycans (PGs);
B, the HS/CS tetrasaccharide linker;
C, glycosyltransferases involved in GAG biosynthesis;
D, genes involved in the structural modification of heparan sulfate (HS);
E, genes involved in the structural modification of chondroitin sulfate (CS);
F, GAG-degrading glycosyl hydrolases.
From left to right, bars in the graphs represent relative transcript abundance in axenic reconstructed corneal epithelia (dark blue), or after exposure to *Pseudomonas aeruginosa* BEVs (orange), *Staphylococcus epidermidis* BEVs (green), lipopolysaccharide (purple), peptidoglycan (dark gray), *lipoteichoic acid* (light gray). Data are plotted on a logarithmic scale for each gene analyzed, and the error bars represent standard deviations. Genes showing statistically significant differences in transcription levels are highlighted.

Supplementary Figure S2

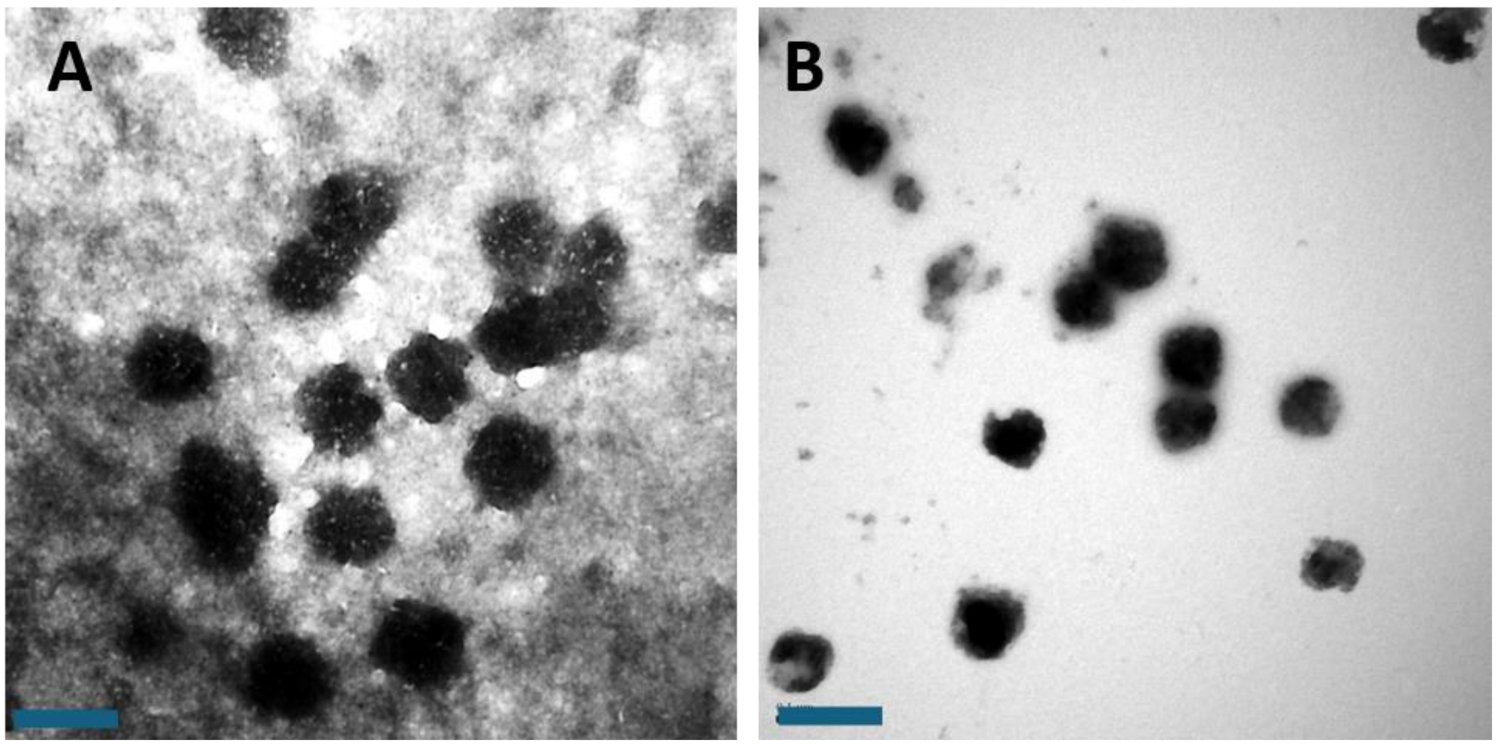

Representative transmission electron microscopy (TEM) images of bacterial extracellular vesicles (BEVs) isolated from *Pseudomonas aeruginosa* (A) and *Staphylococcus epidermidis* (B). Vesicles displayed the expected spherical morphology and nanoscale dimensions.
Scale bar = 200 nm.

Supplementary Table S1. Primer sequences, gene identifiers, and encoded proteins/enzymes used for RT-qPCR analysis.

Gene	Gene ID	Enzyme/product	Primer sequence
SDC1	6382	Syndecan-1	F 5'- CTCAGGTGCAGGTGCTTTG<br>R 5'- CTGCGTGTCCTTCCAAGTG
SDC2	6383	Syndecan-2	F 5'- GATGACGATGACTACGCTTCTG<br>R 5'- TGGAAGTGGTCGAGATGTTG
SDC3	9672	Syndecan-3	F 5'- CTCCTTTCCCGATGATGAAC<br>R 5'- CGACTCCTGCTCGAAGTAGC
SDC4	6385	Syndecan-4	F 5'- GGCAGGAATCTGATGACTTTG<br>R 5'- TCTAGAGGCACCAAGGGATG
GPC1	2817	Glypican-1	F 5'- CATCGGGTGTGGAGAGTG<br>R 5'- TGAGCGTGTCCCTGTTGTC
GPC2	221914	Glypican-2	F 5'- CTGGGACACGACCTGGAC<br>R 5'- GCCATCCAGTCATCTGCATAC
GPC3	2719	Glypican-3	F 5'- CTGCTTCAGTCTGCAAGTATGG<br>R 5'- GTGGAGTCAGGCTTGGGTAG
GPC4	2239	Glypican-4	F 5'- AGTGTGGTCAGCGAACAGTG<br>R 5'- CAAACATATCATTCAGGGATTTCTC
GPC5	2262	Glypican-5	F 5'- GCCGCCCTGTAAGAACAC<br>R 5'- TCATTCCATGCTTCTCTTTGC
GPC6	10082	Glypican-6	F 5'- CCAGGCATAAGAAATTTGACG<br>R 5'- CATGTACAGCATGCCATAGGTC
PRCAN	3339	Perlecan (HSPG2)	F 5'- TGGACACATTCGTACCTTTCTG<br>R 5'- CACTGCCCAGGTCGTCTC
AGRN	375790	Agrin	F 5'- ACTGTGTCTGCCCGATGC<br>R 5'- GACACTCGTTGCCGTATGTG
COL18A1	80781	Collagen XVIII	F 5'- GTACAAGGGAGAGATTGGCTTTC<br>R 5'- TTTCTCTCCTTTCAATCCGTTC
TGFBR3	7049	Betaglycan<br>(TGF-β receptor type III)	F 5'- AGTGTGAGCTGACGCTGTGTA<br>R 5'- GGGCTTAGTGAACGTCTTCTTATTC
CD44 (V3)	960	CD44 hyaluronan receptor	F 5'- TGGGAGCCAAATGAAGAAAATGAA<br>R 5'- TGGTTGAAATGGTGCTGGAGA
SRGN	5552	Serglycin	F 5'- TCCTGGTTCTGGAATCCTCA<br>R 5'- TCTTGTTGGATTCACCTGGAA
NRP1	8829	Neuropilin-1	F 5'- CAACGGGGAAGACTGGATCAC<br>R 5'- AGTTGCAGGCTTGATTCGGA
XYLT1	64131	Xylosyltransferase-1	F 5'- ACTACCCCATCAGGACAAATGA<br>R 5'- CTGCTTCCGAATGAACCTTG
XYLT2	64132	Xylosyltransferase-2	F 5'- AGGGCCTGGTAGTGTGGAG<br>R 5'- TGAACTGTCTGTGTCCTTGGAA
B4GALT7	11285	β-1,4-galactosyltransferase 7	F 5'- GCGAGGACGACGAGTTCTAC<br>R 5'- CAGGTGGCGAAATGTCTTGTA
B3GALT6	126792	β-1,3-galactosyltransferase 6	F 5'- CACGTGGCCTTCGAGTTC<br>R 5'- CCGAGAAGAAGCCCCAGTA
B3GAT2	135152	β-1,3-glucuronyltransferase 2	F 5'- GCTGACGACGACAACACCTA<br>R 5'- CGGTGTACCAGCCAACAAC
B3GAT3	26229	β-1,3-glucuronyltransferase 3 (GlcAT-I)	F 5'- GAAGAACGTGTTTCTCGCCTAC<br>R 5'- CCTCAGATCCTTCTGCCGTA
FAM20B	9917	Xylosylkinase	F 5'- TCTGCAGAAGCACCGTCA<br>R 5'- CAGCTGTGTCAATGATGTCCA
PXYLP1	92370	2-phosphoxylose phosphatase 1	F 5'- AATCATGCCCGACCCTGTG<br>R 5'- TGAACACATGCACTGAGACCA
EXTL1	2134	Exostosin-like glycosyltransferase 1	F 5'- GATGAGAGGCTCCCACTTCA<br>R 5'- CCTCCAGAGTGGTATGGATGA
EXTL2	2135	Exostosin-like glycosyltransferase 2	F 5'- TGAACTGGAAACCAATGCAG

			R 5’- AGGAAATTGCTGCCAAACTG
EXT1	2131	Exostosin-1	F 5’- GAGACAATGATGGGACAGACTTC<br>R 5’- CTCTGTCGCTGGGCAAAG
EXT2	2132	Exostosin-2	F 5’- CTGGGACCATGAGATGAATA<br>R 5’- GATATCCCCAGGCATTTTGTA
CSGALNACT1	55790	Chondroitin sulfate N-acetylgalactosaminyltransferase 1	F 5’- TCAGGGAGATGTGCATTGAG<br>R 5’- AGTTGGCAGCTTTGGAAGTG
CSGALNACT2	55454	Chondroitin sulfate N-acetylgalactosaminyltransferase 2	F 5’- GCCATTGTTTATGCCAACCA<br>R 5’- ATCCACCAATGGTCAGGAAA
CHSY1	22856	Chondroitin sulfate synthase 1	F 5’- GCCCAGAAATACCTGCAGAC<br>R 5’- GCACTACTGGAATTGGTACAGATG
CHPF	79586	Chondroitin polymerizing factor	F 5’- GGTGCACTATAGCCATCTGGA<br>R 5’- GGCACTTCGGAAATGAGG
CHSY3	337876	Chondroitin sulfate synthase 3	F 5’- GACTCAGTGTGTCTGGTCTTACG<br>R 5’- TTGCTATTGTGAAGGTCTTGGA
HAS1	3036	Hyaluronan synthase 1	F 5’- TCCACTGTGTATCCTGCATCAG<br>R 5’- GCCGGTCATCCCCAAAAGTA
HAS2	3037	Hyaluronan synthase 2	F 5’- ACCAAGAGCTGAACAAGATGCA<br>R 5’- GCCAACAATATAAGCAGCTGTGA
HAS3	3038	Hyaluronan synthase 3	F 5’- CGCGCCCCTTCAGCATATG<br>R 5’- CACATAGGCTGCCAGGATGC
NDST1	3340	N-deacetylase/N-sulfotransferase 1	F 5’- CTGCCCTCTACCTGTTCCTG<br>R 5’- AACTGGATCTCCTCAAAGGTCTC
NDST2	8509	N-deacetylase/N-sulfotransferase 2	F 5’- CAAGAGCTGCGTACCAACC<br>R 5’- GAGGGTCCGTGTGTAGTTCAG
NDST3	9348	N-deacetylase/N-sulfotransferase 3	F 5’- CCTTGCAGAAGAGATGTTTGG<br>R 5’- GTAGCAGGATCAGTTCTTAGTTGTTG
NDST4	64579	N-deacetylase/N-sulfotransferase 4	F 5’- GACATTGGGCTCCATCTGAC<br>R 5’- GCTGCTGTCCATCAATAATTAGC
GLCE	26035	D-glucuronyl C5-epimerase	F 5’- TGTGGAAGTCCGAGACAGAG<br>R 5’- CTGGATTGGATAGAAATAGCCTTG
HS2ST1	9653	Heparan sulfate 2-O-sulfotransferase 1	F 5’- TGGAGATGATTATAGACCAGGGTTAC<br>R 5’- GCTATGGCCACAGAAGAACG
HS6ST1	9394	Heparan sulfate 6-O-sulfotransferase 1	F 5’- GCAGGGAGTGGAGCTAACAG<br>R 5’- AACAGTTCCAGTTCCCGAAA
HS6ST2	90161	Heparan sulfate 6-O-sulfotransferase 2	F 5’- CGGTGCGATCTTCTCCAA<br>R 5’- AGGACGATCACGGCAAATAG
HS6ST3	266722	Heparan sulfate 6-O-sulfotransferase 3	F 5’- CAACCACAGCCACACCAG<br>R 5’- CTTCTTCCATCACACATATGAAGAG
HS3ST1	9957	Heparan sulfate 3-O-sulfotransferase 1	F 5’- CAGCCAGATGCCCTTCTC<br>R 5’- AGACTCGCTCAGGCACTTTG
HS3ST2	9956	Heparan sulfate 3-O-sulfotransferase 2	F 5’- GATTGGTACAGGAGCCTGATG<br>R 5’- GGAGCCTCTTGAGTGACAAAG
HS3ST3A1	9955	Heparan sulfate 3-O-sulfotransferase 3A1	F 5’- GGCCGAGAGAACCTGAACTC<br>R 5’- CGAGCGACAGTGACTTCCA
HS3ST3B1	9953	Heparan sulfate 3-O-sulfotransferase 3B1	F 5’- GCAGATCTTGCCTCGATGTC<br>R 5’- GCGCACGAGTACAGGAACATA
HS3ST4	9951	Heparan sulfate 3-O-sulfotransferase 4	F 5’- TAGAGCCGCACTTCTTCGAC<br>R 5’- GGTTATTTGCCCATCCAAAG
HS3ST5	222537	Heparan sulfate 3-O-sulfotransferase 5	F 5’- CATCCGGCAGTAGTCAAAGC<br>R 5’- TTGTGATTTGCTGAGGGTAGG
HS3ST6	64711	Heparan sulfate 3-O-sulfotransferase 6	F 5’- GCCCTGCTGGAGTTTCTG<br>R 5’- GCGCTCGTAGCACCTGTC
SULF1	23213	Extracellular endosulfatase 1	F 5’- CCAGCAGAAGCCAAAGAAAG<br>R 5’- GAACGTGTCTGCCGAGTATG
SULF2	55959	Extracellular endosulfatase 2	F 5’- GCCTGCAAGAGAAGGACAAG<br>R 5’- AGCAGCTTGCGGAGTTTC
CHST11	50515	Chondroitin 4-O-sulfotransferase 1	F 5’- CGCTGCTGGAAGTGATGA<br>R 5’- AGGATAAAGGATCCCAAGCAA
CHST12	55501	Chondroitin 4-O-sulfotransferase 2	F 5’- GTAGCCGACAAATCCTTCCA<br>R 5’- ACCGGTTTACCTCTGACTTGAC

CHST13	166012	Chondroitin 4-O-sulfotransferase 3	F 5’- CCGGCATTTGGAAACAGA<br>R 5’- TCCAGGTCATAGAGCTTCTGC
CHST14	113189	Dermatan 4-O-sulfotransferase 1	F 5’- CCACTGCCTAATGTCACCAA<br>R 5’- ATGACAGGCAGAAGCACAGA
CHST15	51363	GalNAc4S-6-O-sulfotransferase	F 5’- GTGCCAGGAATAAAGTTCAACA<br>R 5’- CACTGGATAAGTCCCGAGTGA
CHST3	9469	Chondroitin 6-O-sulfotransferase 1	F 5’- TGCACAGCCTGAAGATGAGA<br>R 5’- CAGCTTGTCTGAGACCCTTGA
CHST7	56548	Chondroitin 6-O-sulfotransferase 2	F 5’- GATCCGGGTCAGTCACCA<br>R 5’- GACAGATTGCCCCCACAG
DSE	29940	Dermatan sulfate epimerase	F 5’- GTCCAGAGGCACTTCAACATC<br>R 5’- AGTCCGCAATAGCCACAGTC
UST	10090	Uronyl 2-O-sulfotransferase	F 5’- ACCATGGACCACCTCCTAGTAA<br>R 5’- CACACTTGCCTACCCTGTTGTA
HPSE	10855	Heparanase	F 5’- ATGCTCAGTTGCTCCTGGAC<br>R 5’- CTCCTAACTGCGACCCATTG
HPSE2	60495	Heparanase-2	F 5’- GGCCGAGGAAGAATGTCA<br>R 5’- GTGTGTCTAACAGGCGAGTTTTC
HYAL1	3373	Hyaluronidase-1	F 5’- GCTGCCCTATGTCCAGATCTTC<br>R 5’- TGGTTCTTGTATTTTCCCAGCTCA
HYAL2	8692	Hyaluronidase-2	F 5’- TCTACCATTGGCGAGAGTGC<br>R 5’- CCAGCAGCCGTGTCAGGTAATC
HYAL3	8372	Hyaluronidase-3	F 5’- GGATGACCTTGTGCAGTCCA<br>R 5’- AGATGCCAGCACTCCTCCTC
HYAL4	23553	Hyaluronidase-4	F 5’- TGACTAAAGGACCAGCAGCAAA<br>R 5’- CCATGAAGTGAGATGTACTGGTTGA
SPAM1	6677	Hyaluronidase (PH-20)	F 5’- TTTGGCGAAACTGTTGCTCTG<br>R 5’- GCTGCTAGTGTGACGTTGATTATG
